# Gaze-Following and Reaction to an Aversive Social Interaction Have Corresponding Associations with Variation in the OXTR Gene in Dogs but Not in Human Infants

**DOI:** 10.3389/fpsyg.2017.02156

**Published:** 2017-12-12

**Authors:** Katalin Oláh, József Topál, Krisztina Kovács, Anna Kis, Dóra Koller, Soon Young Park, Zsófia Virányi

**Affiliations:** ^1^Research Centre for Natural Sciences, Institute of Cognitive Neuroscience and Psychology, Hungarian Academy of Sciences, Budapest, Hungary; ^2^Department of Cognitive Psychology, Eötvös Loránd University, Budapest, Hungary; ^3^Department of Medical Chemistry, Molecular Biology and Pathobiochemistry, Semmelweis University, Budapest, Hungary; ^4^Comparative Cognition, Messerli Research Institute, University of Veterinary Medicine, Vienna, Austria; ^5^University of Vienna, Medical University of Vienna, Vienna, Austria

**Keywords:** oxytocin, gaze following, social, fear, genotype, dog, human infant

## Abstract

It has been suggested that dogs' remarkable capacity to use human communicative signals lies in their comparable social cognitive skills; however, this view has been questioned recently. The present study investigated associations between oxytocin receptor gene (OXTR) polymorphisms and social behavior in human infants and dogs with the aim to unravel potentially differential mechanisms behind their responsiveness to human gaze. Sixteen-month-old human infants (*N* = 99) and adult Border Collie dogs (*N* = 71) participated in two tasks designed to test (1) their use of gaze-direction as a cue to locate a hidden object, and (2) their reactions to an aversive social interaction (using the still face task for children and a threatening approach task for dogs). Moreover, we obtained DNA samples to analyze associations between single nucleotide polymorphisms (SNP) in the OXTR (dogs: −213AG, −94TC, −74CG, rs8679682, children: rs53576, rs1042778, rs2254298) and behavior. We found that OXTR genotype was significantly associated with reactions to an aversive social interaction both in dogs and children, confirming the anxiolytic effect of oxytocin in both species. In dogs, the genotypes linked to less fearful behavior were associated also with a higher willingness to follow gaze whereas in children, OXTR gene polymorphisms did not affect gaze following success. This pattern of gene-behavior associations suggests that for dogs the two situations are more alike (potentially fear-inducing or competitive) than for human children. This raises the possibility that, in contrast to former studies proposing human-like cooperativeness in dogs, dogs may perceive human gaze in an object-choice task in a more antagonistic manner than children.

## Introduction

Dogs show various forms of strikingly human-like performance at the behavioral level (for a review see Hare and Tomasello, 2005), and this convergence in behavior is most marked in the social contexts that require dogs to interact with humans (Miklósi and Topál, 2013). More specifically, it has been proposed that dogs possess a sensitivity to human communicative cues that parallels that of human children (for a review see Topál et al., 2014). This similarity is likely to have important functions, as arguably, much of the higher-order cognitive skills of humans rest on the fundamental ability to participate in and make use of communicative interactions in a unique way (e.g., Boyd and Richerson, 1998; Csibra and Gergely, 2011). The special receptivity to communicative signals enables the acquisition of generalizable, culture-specific knowledge, ultimately laying the ground for the accumulation of knowledge over generations. Studies with infants have confirmed that humans already at an early age process information presented in an ostensive context in a specific way: they expect this information to be generalizable and not restricted to the given context (e.g., Topál et al., 2008; Futó et al., 2010). For example, Topál et al. (2008) have shown that children, after repeatedly observing that an object is hidden at one location (A), tend to erroneously search for the hidden object in its initial hiding location even after witnessing that the object has been placed in another location (B). This is the case only if the experimenter has addressed them communicatively before hiding the object. If, however, the original hiding event (A) is not accompanied by ostensive communicative cues, the children commit this search error significantly less often. This, in sum, suggests that they interpret the ostensive (but not the non-ostensive) A trials as a learning situation, and generalize the acquired knowledge to the B trials. Similar results were obtained with dogs using the same paradigm (Topál et al., 2009), suggesting comparable sensitivity to human communication in the two species, at least at the behavioral level.

Such remarkable similarities in performance have initially tempted researchers to assume that they reflect human-like social cognition in dogs (Hare et al., 2002). However, more recent rigorous analyses show that different cognitive mechanisms may be at play. Importantly, Topál et al. (2009) compared the performance of dogs and children in a novel condition where a crucial difference in their behavior emerged: while children continued to search in location A following a communicative demonstration even when there was a new social partner present, the change in social context seemed to provide a clean slate for dogs. Thus, the authors conclude that despite the similarities in superficial behavior, the cognitive processes may be markedly different. While children's behavior can be explained by their bias to interpret information as generalizable across contexts, what dogs may extract from such demonstrations is an instruction to produce a certain action, which retains validity as long as the person giving the instruction is present. Later results have confirmed this interpretation suggesting that dogs tend to pick up information from ostensive communication that is restricted to the “here and now” (Sümegi et al., 2014).

Similarly to the above presented account, it took two decades of research to determine the underlying mechanism of dogs' outstanding success in following human pointing (Lakatos et al., 2009). In contrast to early assumptions that dogs, just as children, interpret pointing as a form of cooperative referential communication that offers them food and information where it can be found (Hare and Tomasello, 2005), recent analyses have showed that dogs take pointing as an imperative that sends them to the highlighted location (Kaminski et al., 2012; Tauzin et al., 2015). Children interpret not only pointing but also directional gaze cues as communicative signals that are supposed to provide them with generalizable knowledge (Senju et al., 2008). Dogs have also been believed to use human gaze similarly to pointing, often ignoring findings that even after a communicative gaze cue, dogs choose one of two food locations randomly (Kaminski et al., 2012). Differences in the two species' reactions to directional cues have been further highlighted by recent studies showing that while children ignore gaze cues in a non-ostensive context (Senju and Csibra, 2008), dogs actually avoid food locations indicated with non-communicative gaze by both a conspecific and a human (Bálint et al., 2015; Duranton et al., 2017). Based on these findings, it has been hypothesized that dogs see an object choice task with non-communicative gaze as food competition and they tend to behave in a way that can help to avoid a potential conflict over food (Duranton et al., 2017). Interestingly, it appears that oxytocin can mitigate this effect: Oliva et al. (2015) found that after intranasal oxytocin administration dogs were more likely to choose the one of two food containers that had been indicated with a human gaze cue.

While oxytocin seems to facilitate social approach and social cognition in general both in dogs and humans (Bartz et al., 2011; for reviews see Kis et al., 2017), one of the best described mechanisms behind these facilitation effects is related to the attenuation of fear responses and anxiety. Most likely contributing to such effects, oxytocin has been shown to inhibit the responsiveness of the hypothalamo-pituitary-adrenal axis (Neumann, 2002) as well as to attenuate the activity of the amygdala in response to both threatening (Huber et al., 2005) and positive stimuli (Domes et al., 2007). Not only oxytocin production but also oxytocin receptor binding is a key component of the oxytocinergic system. In line with this, increasing evidence suggests that genetic polymorphisms of the oxytocin receptor gene (OXTR) also play a role in modulating various behaviors in social interactions, ranging from fearful behaviors through emotion processing to prosociality (see below). A number of studies have looked at associations between human social behavior and different single nucleotide polymorphisms (SNP) in the OXTR, and a few SNPs have emerged as having a prominent role in shaping socio-cognitive skills and social behavior. The OXTR rs53576 polymorphism (in intron 3) is probably the most intensively investigated SNP (for a meta-analysis, see Li et al., 2015), and it has been associated with—among others—stress reactivity (Rodrigues et al., 2009), need for social support (Kim et al., 2010) and emotion processing (Tost et al., 2010). The rs2254298 polymorphisms (in intron 3) in the OXTR have been linked to attachment anxiety (Chen and Johnson, 2012) and depression (Thompson et al., 2011) in certain populations. A third SNP, rs1042778 (in exon 4 3′ UTR) has also been linked to the regulation of social interactions, in particular by modulating prosocial behavior (Israel et al., 2009). The OXTR SNPs that may account for the variability in the social behavior of dogs are less well known. A few studies have used genetic sequencing to identify loci where significant variations are exhibited between individuals. Kis et al. (2014) have found some SNPs (rs8679682, −212AG, 19131AG) that are associated with proximity seeking and friendliness in dogs. Such variations have been shown to be associated with behavioral differences between dogs and wolves as well, although these loci were not related to within-species behavioral variation (Oliva et al., 2016).

The positive effect of oxytocin on following gaze cues to locate hidden objects may be exerted through at least two mechanisms: either through the reduction of fear (Kirsch et al., 2005; Ring et al., 2006) or through the enhancement of trust (Kirsch, 2015, although see Nave et al., 2015 for the controversy regarding the role of oxytocin in human trust). That is, oxytocin may help to highlight the cooperative aspect of gazing (that is, its perception as an offer of food and information) or it may facilitate approach by reducing fear *despite* the fact that the context remains perceived as competitive. In the present study, following up on recent results described earlier, we set out to test the hypothesis that dogs perceive non-communicative gaze in an object choice task differently to children's interpretation of communicative gaze. More specifically, we aimed to investigate whether OXTR polymorphisms are associated with following human gaze cues in both children and dogs, and if yes whether these associations co-vary with associations the OXTR polymorphisms have with reactions of both species to negative social stimuli. First of all, we hypothesized that the oxytocin system would be related to the modulation of reactions shown in an aversive social context in both species. To test this hypothesis, we used well-established paradigms in both species that have already been shown to evoke distress in participants by violating the expectations of regular adult-infant or human-dog interactions (still face and threatening approach paradigms, respectively). Our second hypothesis was that in dogs, oxytocin would also be related to following of non-ostensive human gaze through the same anxiolytic effect. That is, we predicted that the same OXTR genotypes will be associated with a less fearful reaction to social threat and with higher readiness to follow someone's gaze when searching for food. In contrast, in children, as they do not interpret gaze cues as a threat or competition, we predicted that following gaze will not be associated with OXTR polymorphisms or, if yes, different genotypes will be associated with gaze following and with reactions to a negative social situation. In order to test these hypotheses, we observed (1) the behavior of both infants and dogs in a social context in which their human partner showed negative social behavior unexpectedly, (2) the reaction of infants to communicative gaze, and (3) the reaction of dogs to non-communicative gaze. In addition, buccal samples were obtained from both children and dogs, in order to analyze the associations between behavior and their OXTR polymorphisms located in the intronic as well as the UTR regions of the gene.

## Method

### Participants

#### Human participants

Ninety nine toddlers of 15–16 months participated in the study (mean age: 15.73 months; SD: 0.26 months; range: 15.13–16.2 months). Children were selected from a database of families that had previously indicated interest in participating in research studies and were contacted again for this particular study. An additional 19 children were tested, but excluded from the sample due to fussiness (2), missing or insufficient DNA sample (14) or camera failure (3). 48 out of the 99 toddlers successfully completed both tasks; 28 children met the predetermined criteria only for *the gaze following task* and 16 only for the *still face task* (for more details see Procedure). In total, 76 child participants (36 boys/40 girls) were included in the *gaze following* task and 64 (32 boys/32 girls) in the *still face* task (Table 1). Experiments with children were conducted at the Institute of Psychology, Hungarian Academy of Sciences, Budapest.

**Table 1:** Number of dogs (males/females) and children (boys/girls) included in the different analyses.

**CHILDREN**				
	**Candidate SNP**			
	**rs1042778**	**rs2254298**	**rs53576**	
Gaze following task	76 (36/40)	76 (36/40)	76 (36/40)	
Still face task	64 (32/32)	64 (32/32)	64 (32/32)	
**DOGS**				
	**rs8679682**	−**213AG**	−**94CT**	−**74GC**
Gaze following task	56 (27/29)	51(24/27)	56 (27/29)	48 (24/24)
Threatening approach	56 (30/26)	50 (26/24)	56 (30/26)	48 (27/21)

#### Dog subjects

Seventy one privately owned adult (older than 10 months) Border Collies (mean age: 4.27 years, SD: 2.88 years, 38 females) were recruited and tested at the Clever Dog Lab, Vienna, Austria. Out of the 71 dogs tested, 22 were castrated or spayed (12 females). An additional 5 dogs were tested, but excluded from analyses due to missing or insufficient DNA sample.

Children and dogs that could not be tested with one of the experimental tasks but provided valid data for the other were only excluded from analyses of the specific task in which they failed to participate. Similarly, if DNA could not be sequenced at a given SNP but there was valid data on the other SNPs, the participant was only excluded from the corresponding analyses (Table 1 shows the number of dogs and children that were included in each analyses out of the 71 subjects and 99 participants, respectively).

### Ethics statement

The study with child participants was approved by the United Ethical Review Committee for Research in Psychology (Ref No. XIV-I-001/531-4-2012). For dog participants, ethical approval was obtained in accordance with GPS (Good Practice Statement) guidelines and national legislation by the Ethical Committee for the use of animals in experiments at the University of Veterinary Medicine Vienna (Ref No. 04/12/97/2012). Participants' owners (dogs) or caregivers (children) signed informed consent prior to participation.

### Procedure

Both children and dogs took part in two tests. *Task 1* was construed to test their sensitivity to a human gaze cue. *Task 2* was construed to assess their reaction to an aversive social interaction with a human experimenter. Testing was conducted by two female experimenters for children and three female experimenters for dogs. In order to standardize their behavior, all experimenters received a detailed experimental protocol and watched the other experimenter(s) conducting the tests. The next sections describe the species-specific testing situations separately.

#### Task 1: following a human gaze cue

##### children

*Familiarization trials* Prior to the experiment, children engaged in playful activities together with their mothers and the experimenter in order to familiarize them with the environment (10 min).

*Test trials* Children were seated on their caregivers' lap on a 50 cm high chair. Parents were instructed to hold their children on their laps or were allowed to let children stand on the ground while the parent was holding them at a fixed position. The experimenter kneeled on the floor about 2 meters away from the child and the parent, facing them. She presented two identical opaque boxes to the participant, placing them in front of her 60 cm apart from each other. Once the child's attention was engaged, she opened the two boxes (starting always with the one on her left), revealing that one of the boxes contained a small toy. To make sure children realized the toy in the box, the experimenter lifted the boxes, moved closer to the participant and showed them the content of the boxes close up. During this procedure, she communicated with the child in a natural manner, which included calling the child's name, using attractive facial expressions and engaging in eye-contact repeatedly. After that, she placed the boxes back at their original locations and closed the lids, starting with the one on the left. Then, she switched the location of the boxes three times in view of the child, but with a relatively fast motion in order to confuse children about the location of the baited box. This way, the baited box ended up on the opposite side of the experimenter. The experimenter then looked up at the child in order to initiate eye-contact with them. Once the child was engaged in eye-contact, the experimenter called their name and turned her head toward the baited box and kept looking at it. After 5 s had elapsed, she turned her gaze back toward the child, smiling. At this point, parents (as a priori instructed) let go of their children, and participants were allowed to approach the boxes and look for the toy. If children touched one of the boxes or clearly pointed at one, the test was terminated and the experimenter helped open the box, revealing its content to the child. If children did not make a choice in the first 60 s they were coded as passive and were excluded from analyses.

##### Dogs

*Familiarization trials* This phase was included to familiarize the dogs with two small containers (10 cm diameter, 15 cm height). Before the start of the trial, the experimenter placed the two containers on the floor randomly, but about 1.5 m apart from each other, baiting only one of them with food (a small piece of cheese or sausage). The owner then let the dog free to enter the experimental room to search the two containers and eat the food, and waited with the experimenter outside of the room with the door open. If the dog did not start searching within 30 s after being released, the owner entered the room and encouraged the dog to search. A trial ended once the dog ate the food. In total, there were 4 familiarization trials.

*Test trial* Before the test trial began, the experimenter placed the two containers, in the same way as in the familiarization trials, and a chair at an equal distance of about 2 m from the two containers. The experimenter kneeled between the two containers and waited, keeping her hands behind her back and looking straight ahead. The test began as the owner and the dog entered the room. The owner sat on the chair, keeping the dog on a short leash so that the dog could not approach the containers closer than 1 m. Once the owner sat down on the chair, the experimenter waited to make eye contact with the dog. If the experimenter was not able to do so within 10 s, she tried to get the attention of the dog by calling its name, but minimized other communication. As soon as eye contact was established, the experimenter kept looking into the dogs' eyes with a blank facial expression while staying still and silent. Once the dog broke the eye contact, the experimenter called the dog's name and made another brief eye contact and, with a clear head movement, turned her head to look down at the baited container. After 5 s had elapsed, the owner released the dog to choose a container, while the experimenter was still looking at the baited container. The trial ended when the dog touched one of the containers with its mouth.

#### Task 2: reaction to an aversive social interaction

The second task was designed to describe how the participants reacted in a socially aversive situation. As our goal was not to directly compare the behavior of children and dogs but to compare the behavioral associations of the OXTR SNPs across tasks and within species, we chose slightly different paradigms that detect individual variation both in children and dogs (*still face* task, Tronick et al., 1978 and *threatening approach* task, Vas et al., 2005, respectively). Both tasks have been described to evoke distress and frustration in participants through the violation of expectations of regular adult-infant or human-dog interactions. In the *still face* task, this is achieved by the withdrawal of the experimenter's communication and her lack of reactivity. In the *threatening approach* task, the prolongation of her approach and her intense looking evoke this mismatch. Importantly, children and dogs have also been described to show a similar range of reactions to these situations: some try to repair this mismatch by attempting to engage the partner in friendly interactions, some attempt to leave the unpleasant social situation, whereas others exhibit signs of distress or frustration as a response to the violation of the expected social behavior (Tronick et al., 1978; Vas et al., 2005).

##### Children: Still face

Children participated in the *still face* task (c.f. Tronick et al., 1978) to test their reactions to the withdrawal of positive social stimulation from the experimenter. The task consisted of two 1-min-long phases. The caregiver was instructed to take a seat on one side of a 1.5 m long blanket and hold their child on their lap. The experimenter sat down at the other end of the blanket, facing the child. In the first phase of the task, the experimenter engaged the child in a session of peek-a-boo game, where she alternated between initiating eye-contact with the child (smiling) and hiding her face behind a veil or her hands. After 1 min had elapsed, a second experimenter signaled the start of the second phase. To ostensibly separate the two phases, upon hearing the signal from the second experimenter, the first experimenter turned her head away from the child and when she looked back, she began the still face phase, during which she was silently looking at the child but did not initiate any further contact and did not respond to the child's attempt to communicate. After 1 min had passed, the test phase ended and the experimenter resolved the possible negative feelings caused by the still face episode by starting the peek-a-boo game again.

##### Dogs: threatening approach

The test procedure was similar to the procedure described in the experiment of Vas et al. (2005) in which the dog's response to the unexpected threatening behavior of the experimenter was recorded. The dog was on a leash fixed on a wall in the room, while the owner was standing about 30 cm behind the dog. The experimenter, who had previously interacted with the dog and its owner in a friendly manner, entered the room from the side door and stood about 5 m away from the dog. Once the dog looked at her, the experimenter started to approach the dog slowly (one step in every 4 s) with her upper body slightly bent and looking steadily into the eyes of the dog without any verbal communication.

The behavior of the experimenter was determined and standardized across subjects according to the following rules: (1) If the dog kept looking at the experimenter without any other reaction, then she continued to approach the dog until she reached it. (2) If the dog broke the eye contact with her (moving away and/or turning head away), the experimenter stopped and waited motionless for about 4 s and then tried to attract the dogs attention by making some noise (a slight cough or scratching the ground with the foot). If the dog continued to avert his gaze, the experimenter attempted to call the dog's attention two more times (with 2 s in between attempts). Whenever the dog looked at her again, she continued the approach. If, however, the dog did not look at her after the third attempt, the test was terminated. (3) If the dog showed active avoidance, that is, moved behind the owner, the test was immediately terminated. (4) If the dog showed signs of aggression, e.g., barked repeatedly or growled continuously (longer than 4 s) and/or tried to attack the experimenter, the test was terminated. If the subject did not show any form of fear or aggression even when the experimenter reached the dog, she touched the dog's head and gently petted it.

### Buccal sample collection and SNP genotyping

Buccal cell samples were collected from each participating dog and child by swabbing the upper gum area with 4 cotton swabs. The cotton swabs were then sealed in a tube and preserved in the freezer until genotyping (Bence et al., 2017). DNA purification was initiated by incubating the buccal samples at 56°C overnight in 0.2 mg/ml Proteinase K cell lysis buffer. It was followed by protein denaturation using saturated NaCl solution. Finally, DNA was precipitated using isopropanol and ethanol by standard procedures and DNA pellet was resuspended in 100 μl 0.5 × TE (1 × TE: 10 mM Tris pH = 8, 1 mM EDTA) buffer.

For both species, we genotyped polymorphisms that had been linked to social behavior in former studies. For infants, these were the SNPs rs1042778; rs2254298 and rs53576 (based on Israel et al., 2009; Rodrigues et al., 2009; Chen and Johnson, 2012; for instance). For dogs SNPs −213AG; −74CG; −94TC; and rs8679682 were genotyped (Bence et al., 2017). Note that these SNPs, although all in the OXTR gene, are neither structurally nor functionally equal between dogs and humans.

#### Dogs

Typical DNA concentration of the dogs' genomic DNA samples isolated from buccal swabs was around 20 ng/μl. The Qiagen Hot-StarTaq polymerase kit was used for PCR amplification. The reaction mixture contained 1 μM of each primer, approximately 5 ng of DNA template, 200 μM dNTP, 0.025 U HotStarTaq DNA polymerase, 1 × buffer, and 1 × Q-solution supplied together with the enzyme. The PCR cycle consisted of an initial denaturation at 95°C for 15 min, 40 cycles of 1-min denaturation at 95°C, 1-min annealing at various temperatures, a 1-min extension at 72°C, and a 10-min final extension at 72°C. The PCR reaction was performed in a total volume of 10 μl. −213AG and the −74CG polymorphisms were genotyped by PCR-RFLP method. PCR amplification was performed as described above using 5′-CCA TTG GAA TCC GCC CCC T-3′ forward and 5′- CAC CAC CAG GTC GGC TAT G-3′ reverse primers. Annealing temperature was 56°C. PCR products were incubated for 3 h at 37°C in a restriction enzyme mixture containing 0.5 U/μl Hpy99I restriction enzyme (NEB) for −213 SNP and 0.5 U/μl BsiEI restriction enzyme (NEB) for −74CG SNP, 1xBSA and 1x NEB4 buffer. Total reaction volume was 16 ml −94TC SNP was genotyped by allelespecific amplification (ASA) using the primers described above. Allele specific primers were 5′-CCG ATC TGC TGG TCC CGG-3′ and 5′-CCG ATC TGC TGG TCC CGA-3′ and the annealing temperature was 60°C. rs8679682 SNP was genotyped by real-time PCR using sequence specific TaqMan probes with minor groove binding (MGB) quencher. Primers were designed by Primer Express 3.0 (forward primer: 59-CTC CTT TAT TTTGGG ATC TTG TGA A-39, reverse primer: 59-CCT GCT CCTTAT TCT GAG CTT AGA A-39, probe specific for T allele: 59-FAM-AGT GGT AAG TAT AGG ATT G-MGB-39, probe specific for A allel: 59-VIC-AGT GGT AAG TAA AGG ATMGB-39.

The PCR products were analyzed by conventional submarine agarose gel electrophoresis (Biocenter, Szeged, Hungary), using 2.5% agarose gel and visualized by ethidium bromide staining. We investigated frequencies and Hardy–Weinberg Equilibrium analyses of the genotypes. Allele frequencies (Table 2) did not deviate significantly from the Hardy-Weinberg equilibrium (*p* > 0.05; Chi-square tests). We also tested whether there were any differences in allele frequencies across dogs tested by Experimenter 1, 2 and 3, and found no significant effects (*p* > 0.05; Chi-square tests; see Table 3).

**Table 2:** Allele frequencies for all dogs as well as the number of dogs by task and experimenter.

**Genotype**	**TT**	**CT**	**CC**	**GG**	**AG**	**AA**	**CC**	**CT**	**TT**	**GG**	**CG**	**CC**
Frequency	0.257	0.60	0.143	0.657	0.20	0.143	0.114	0.571	0.314	0.528	0.257	0.1
**GAZE FOLLOWING**												
E1	9	13	3	15	6	2	3	12	10	13	5	3
E2	1	11	4	12	3	0	2	7	7	9	3	2
E3	6	8	1	10	2	1	1	11	3	9	3	1
Σ	16	32	8	37	11	3	6	30	20	31	11	6
**THREATENING APPROACH**												
E1	10	12	3	15	5	2	2	13	10	13	5	3
E2	1	12	4	13	3	0	3	8	6	8	5	2
E3	5	8	1	9	2	1	1	10	3	8	3	1
Σ	16	32	8	37	10	3	6	31	19	29	13	6

**Table 3:** Allele frequencies for all children and the number of children by task and experimenter.

**Genotype**	**rs1042778**			**rs2254298**			**rs53576**		
	**TT**	**TG**	**GG**	**GG**	**AG**	**AA**	**GG**	**GA**	**AA**
Frequency	0.151	0.353	0.496	0.777	0.222	0	0.374	0.444	0.182
**GAZE FOLLOWING**									
E1	8	20	25	42	11	0	23	22	8
E2	5	6	12	19	4	0	8	9	6
Σ	13	26	37	61	15	0	31	31	14
**STILL FACE**									
E1	7	16	21	36	8	0	19	19	6
E2	2	6	12	15	5	0	8	8	4
Σ	9	22	33	51	13	0	27	27	10

#### Children

Six PCR amplification was performed as described above using 5′- ACT GGG GCA ACC AAA CAT CT-3′ forward and 5′- ACT CTT CAT GGC CCA GAG TG-3′ reverse (rs53576), 5′- GCT CCA GCC AGA GGA G-3′ forward and 5′-AGT GGG TTC AGG GTG GTA-3′ reverse (rs1042778), 5′- CTG TCT TTG CAC CTT TGC TA-3′ forward and 5′- ATG AAA GCA GAG GTT GTG TG-3′ reverse (rs2254298) primers. Annealing temperatures were 56°C (rs53576 and rs2254298) and 60°C (rs1042778). OXTR rs53576 and rs2254298 SNPs were genotyped by PCR-RFLP method. PCR products were incubated for 3 h at 37°C in a restriction enzyme mixture containing 0.5 U/μl AvaII restriction enzyme (NEB) for rs53576 SNP and 0.5 U/μl DdeI restriction enzyme (NEB) for rs2254298 SNP, 1x BSA and 1x NEB4 buffer. rs1042778 SNP was genotyped by allele specific amplification (ASA) using 5′- AGC CAC CCC AAG GAG T-3′ forward and 5′- AGC CAC CCC AAG GAG G-3′ allele specific primers. The PCR products were analyzed by conventional submarine agarose gel electrophoresis (Biocenter, Szeged, Hungary), using 2.5% agarose gel and visualized by ethidium bromide staining. We investigated frequencies and Hardy–Weinberg Equilibrium analyses of the genotypes. Allele frequencies (Table 3) did not deviate significantly from the Hardy-Weinberg equilibrium (*p* > 0.05; Chi-square tests). We also tested whether there were any differences in allele frequencies between children tested by Experimenter 1 and Experimenter 2 and found no significant effects (*p* > 0.05; Chi-square tests; see Table 4).

**Table 4:** Summary of the results.

		**Human**			**Dog**			
		**rs.576**	**rs.778**	**rs.298**	**rs.682**	**−213AG**	**−94TC**	**−74GC**
Gaze following	Main effects	–	–	–	–	**G**	**G**	**G**
	Interactions	–	–	–	–	–	–	–
Still face/Threatening approach look at Caregiver/owner	Main effects	–	–	–	–	–	**G;E**	–
	Interactions	–	–	–	–	–	**S**×**E; S**×**G**;**E**×**G;**	–
Still face/Threatening approach look at Experimenter/Stranger	Main effects	**E**	–	–	–	–		–
	Interactions	–	–	–	**S**×**E;**	–	S × E	–
Threatening approach first reaction	Main effects	*N/A*	*N/A*	*N/A*	**A;S**	**S;G**	–	**G**
	Interactions	*N/A*	*N/A*	*N/A*	–	–	–	–
Still face signs of distress	Main effects	**G;A**	**A; S**	**A**	*N/A*	*N/A*	*N/A*	*N/A*
	Interactions	**E**×**G**	**S**×**E**	–	*N/A*	*N/A*	*N/A*	*N/A*

*G, Genotype; A, Age; E, Experimenter; S, Sex Significant effects are bold, while marginally significant effects are indicated by normal font types. N/A, Not applicable*.

### Data analyses

Behavioral tests were coded offline from the recordings for pre-defined variables. For the *gaze following* task, we coded whether the participants chose the container that had been indicated by the gaze direction of the experimenter. Participants that did not choose a container in the first 90 s were excluded from this part of the analyses. Both for dogs and children, we coded a correct choice if they chose the indicated container.

For the *reaction to an aversive social interaction* task, slightly different measures were used for infants and dogs due to the differences in the procedures. For children, we coded looking times, with a special interest in how much time they spent looking at the experimenter and their caregiver during the still-face period (coding categories: looking at experimenter, looking at caregiver, looking elsewhere). We also coded signs of distress (crying, negative vocalization or negative facial expressions) in the still-face phase. All of the variables were expressed in percentage of time as there could have been slight variations in the total duration times across participants. Infants who left their caregivers' laps during the still face period and spent more than 30% of the time outside of the testing context (that is, were not sitting on the caregiver's lap and were not within a 1 m radius of the experimenter) were excluded from this part of the analyses (*N* = 21). Participants who left the caregiver's lap during the warm-up phase were excluded from all analyses (*N* = 14).

For dogs, we also coded looking times during the *threatening approach* task (looking at the experimenter, the owner or elsewhere). Further on, we coded the dogs' first reaction to the threatening approach of the stranger with the following options: (1), friendly reaction to experimenter (tail wagging while moving toward the experimenter); (2), unfriendly reaction to the experimenter (looking at or approaching experimenter without wagging). Dogs that exhibited extreme stress were excluded from analyses (*N* = 14).

Statistical analyses were performed using SPSS 20.0. Based on the type of the dependent variable (behavioral measures), the associations between genotype and behavior were analyzed using either General Linear Models (Univariate ANCOVA for durations); Binary Logistic Regression (for choice of container and first reaction in the threatening approach task for dogs). We used separate models for each SNPs, and in the ANCOVAs we included age as a covariant, sex (male vs. female), experimenter (2 for children and 3 for dogs) and their two-way interactions both with each other and genotype (3 levels in all cases) in all models. For the regression analyses, we applied a backward elimination method of non-significant effects.

Finally, we also tested whether performance on one test was associated with performance on the other. Thus, we used independent samples *T*-tests to compare behaviors in the *reaction to an aversive social interaction* task between participants that chose correctly vs. incorrectly in the *gaze following* task.

## Results

### Gaze following

#### Children

All 76 children made a choice in this task. Out of the 76 children, 30 chose the baited container (thus, used the gaze direction of the experimenter as a cue to find the hidden object). This does not differ significantly from choosing randomly (though shows a marginal below chance effect) (binomial: *p* = 0.085).

**Figure 1:**
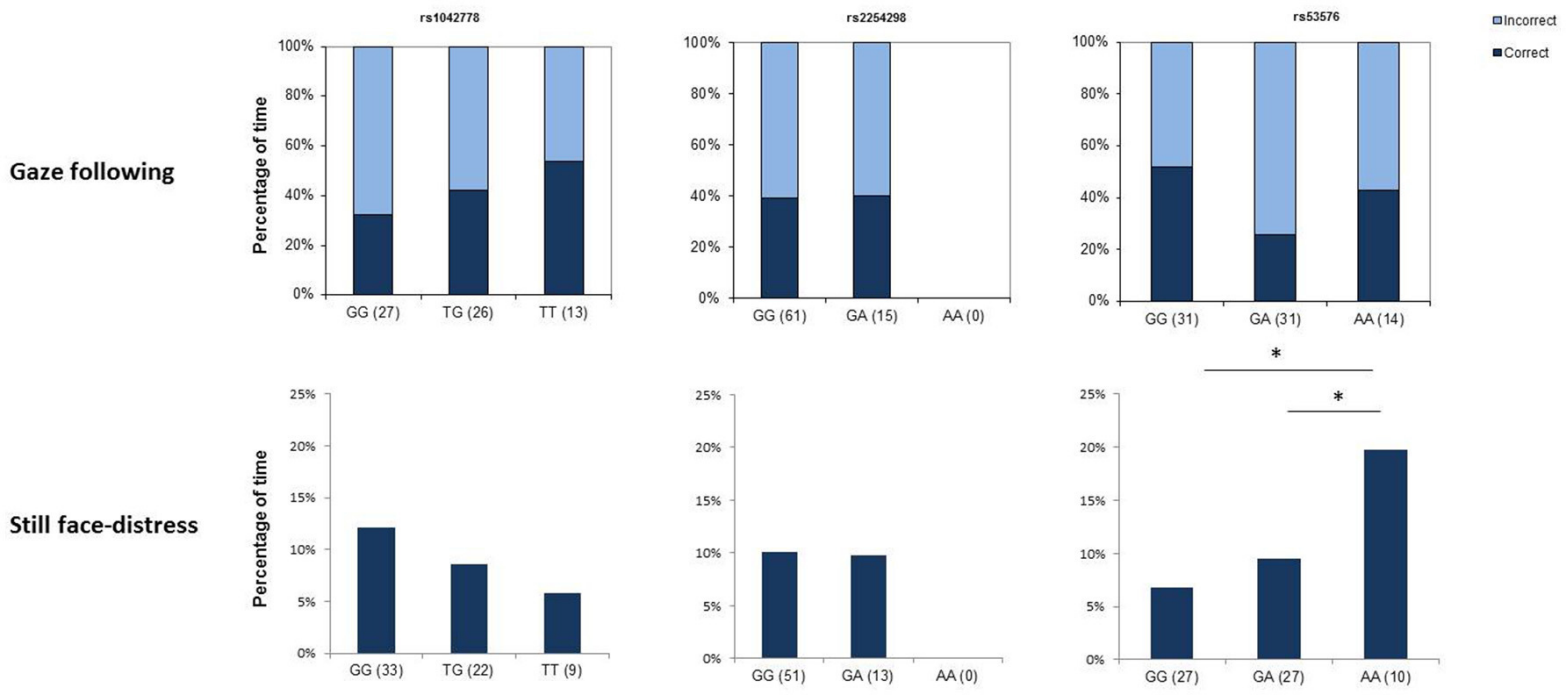
Genotype × behavior associations in children. The upper row shows associations between genotype at the three selected SNPs and success in using communicative gaze as a cue to locate a hidden object, while the lower row depicts associations with the amount of time spent with showing distress signals in an unpleasant social situation (*still face* task). Asterisks mark significant gene × behavior correspondences.

The SNP rs1042778 did not have a significant effect on children's choices and none of the control variables (age, sex and experimenter) did so either (all *p* > 0.283 at removal) (Figure 1). Similarly, we did not find any significant main or interaction effects in the analyses on SNP rs2254298 (all *p* > 0.283 at removal) and SNP rs53576 (all *p* > 0.283 at removal).

#### Dogs

Altogether 57 dogs were included in the sample that both made a choice and had at least one identifiable SNP. Out of the 57 dogs, 38 chose the baited container (thus were successful in using gaze direction as a cue), which does not significantly differ from chance (binomial: *p* = 0.111)

**Figure 2:**
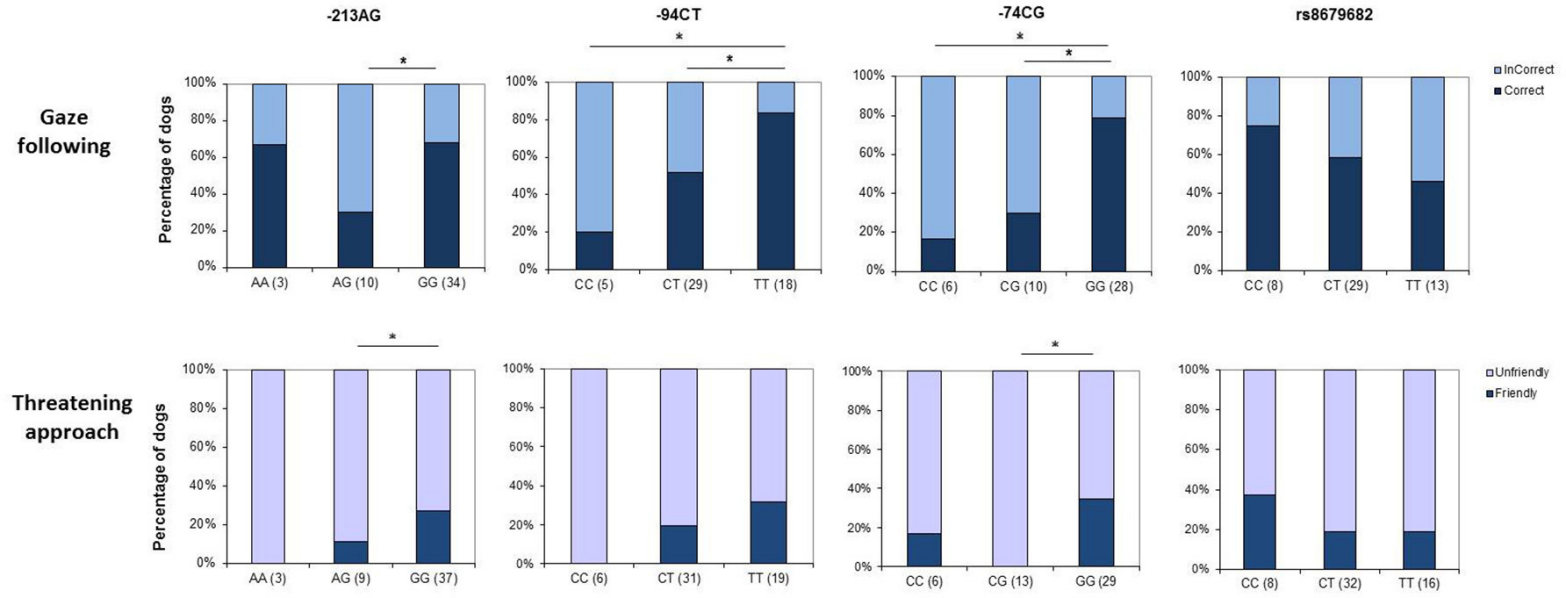
Genotype × behavior associations in dogs. The upper row shows associations between genotype at the four selected SNPs and success in using non-communicative gaze as a cue to locate a hidden object, while the lower row depicts associations with reactions to social threat (*threatening approach* task). Asterisks mark significant gene × behavior correspondences.

The rs8679682 polymorphism did not have a significant main effect on dogs' choices of container [$\chi_{\left(2\right)}^{2}$ = 0.754, *p* = 0.449], and the analyses did not yield any significant effects of the control variables or interaction effects either (all *p* > 0.195 at removal) (Figure 2).

The −94TC polymorphism, however, had a significant effect on dogs' choices of container [$\chi_{\left(2\right)}^{2}$ = 8.267; *p* = 0.016], showing that while dogs with the homozygous C and the heterozygous genotypes made their choices at random, dogs with the homozygous T genotype chose the baited container more often. All other effects were not significant (all *p* > 0.222 at removal).

We also found a marginally significant effect on dogs' choices by the −213AG polymorphism [$\chi_{\left(2\right)}^{2}$ = 5.948; *p* = 0.051]. Dogs with the homozygous G genotype were more likely to follow the correct, baited container; however, this was not true either of the homozygous A or the heterozygous genotypes. All other effects were not significant (all *p* > 0.066 at removal).

SNP −74GC also had a significant effect on dogs' behavior in the task [$\chi_{\left(2\right)}^{2}$ = 13.21; *p* = 0.001], showing that dogs with the homozygous G genotype were most likely to choose the baited container compared to the homozygous A or the heterozygous genotypes. All other effects were not significant (all *p* > 0.109 at removal).

#### Reaction to an aversive social interaction

##### Children—looking at the Experimenter

Allele variations at rs1042778 had no significant effect on the amount of time children spent looking at the experimenter during the still face phase [*F*_(2)_ = 0.39; *p* = 0.679], nor did the analyses yield any significant interaction effects (all *p* > 0.319). Similarly, no main [*F*_(2)_ = 0.065; *p* = 0.8] or interaction effects were found involving SNP rs2254298 (all *p* > 0.15). The model including the SNP rs53576 yielded a significant effect of experimenter [*F*_(1)_ = 4.057; *p* = 0.049], but no main effect of allele variations [*F*_(2)_ = 1.478; *p* = 0.238] and no interaction effects (all *p* > 0.231). Children spent more time looking at Experimenter 2 than at Experimenter 1 [M(E1) = 25.4; M(E2) = 34.24].

##### Dogs—looking at the experimenter

SNP rs8679682 had no significant main effect on the amount of time dogs spent looking at the threateningly approaching experimenter [*F*_(2)_ = 0.607; *p* = 0.55]. However, there was a significant two-way interaction between sex and experimenter [*F*_(1, 54)_ = 4.578; *p* = 0.017], showing that whereas females reacted differently to the two experimenters, males did not. Allele variations at SNP−213AG had no main effect on the time dogs spent looking at the experimenter [*F*_(2)_ = 1.048; *p* = 0.362], and we did not find any significant main effect of the control variables, nor any interaction effects (all *p* > 0.301).

Analyzing the effects of variations at SNP−94TC, we found a marginal effect of allele variation[*F*_(2)_ = 2.647; *p* = 0.084] and a marginal interaction between sex and experimenter [*F*_(1, 54)_ = 2.599; *p* = 0.087]. Results indicate that males differentiated more between experimenters than females, and dogs with the homozygous T genotype spent less time looking at the experimenter than the other two genotypes [M(TT) = 81.06%; M(CT) = 93.11%; M(CC) = 91.39%].

Analyses of the SNP −74GC yielded no main effect of allele variation [*F*_(2)_ = 0.783; *p* = 0.466] and no other effects (all *p* > 0.364).

##### Children—looking at the caregiver

The SNP rs1042778 did not have a significant effect on the amount of time children spent looking at their caregivers [*F*_(2)_ = 0.2; *p* = 0.146]. The interactions involving rs1042778 were not significant either (all *p* > 0.151). Similarly, variations at SNP rs2254298 did not significantly modulate gazing at the caregiver [*F*_(2)_ = 0.002; *p* = 0.965] and the interaction effects were not significant either (all *p* > 0.139). The same was true for SNP rs53576 [main effect: *F*_(2)_ = 0.165; *p* = 0.849; interaction effects: all *p* > 0.296].

##### Dogs—looking at the owner

Rs8679682 had no main effect on the amount of time dogs spent looking back at their owners [*F*_(2)_ = 0.11; *p* = 0.896] and there were no significant main effects of the control variables and no significant interactions either (all *p* > 0.154). Similarly, no effects were found analyzing either SNP −213AG [main effect: *F*_(2)_ = 0.034; *p* = 0.967; other effects: all *p* > 0.377] and SNP −74GC [main effect: *F*_(2)_ = 1.396; *p* = 0.263; other effects: all *p* > 0.523].

However, allele variations at SNP −94TC had a significant effect on dogs' looking times at their owners [*F*_(2)_ = 3.446; *p* = 0.042] and the analyses also yielded a main effect of experimenter [*F*_(2)_ = 6.014; *p* = 0.005], showing that the amount of time spent looking at the owner differed as a function of who was administering the test. These effects were qualified by significant two-way interactions between sex and experiment [*F*_(2, 54)_ = 4.675; *p* = 0.015]; sex and allele variations [*F*_(2, 54)_ = 3.673; *p* = 0.035]; experimenter and allele variations [*F*_(4, 54)_ = 4.913; *p* = 0.003] and a three-way interaction between sex, experimenter and allele variations [*F*_(2, 54)_ = 6.355; *p* = 0.004]. Results show greater variability in the case of males than females. Specifically, looking times increased when Experimenter 2 was administering the test for male dogs with the homozygous C genotype compared to all other cases (*M* = 19.55%, all other Ms<7%.)

##### Children—signs of distress

The analyses on the effects of SNP rs1042778 yielded no main effect of genotype [*F*_(2)_ = 1.579; *p* = 0.216] and no interaction effects involving rs1042778 (all *p* > 0.102) (Figure 1). However, age and sex had marginal effects on the amount of time children exhibited signs of distress [sex: *F*_(1)_ = 3.781; *p* = 0.057; age: *F*_(1)_ = 3.722; *p* = 0.059] and the interaction between sex and experimenter was significant [*F*_(1, 63)_ = 4.555; *p* = 0.038]. The results indicate that younger children exhibited more signs of distress than did older children and boys exhibited more distress than girls [M(girls) = 8.48% of the total duration of the phase; M(boys) = 11.47%]. The interaction shows that there was not a considerable difference in the amount of distress signals in the case of girls [M(E1) = 8.069%; M(E2) = 7.934%]; however boys showed more signs of distress when the test was administered by Experimenter 2 [M(E1) = 8.147%; M(E2) = 21.51%].

In the analyses involving SNP rs2254298, we replicated the connection between age and distress signals [*F*_(1)_ = 5.208; *p* = 0.026], but we found no main effect of genotype [*F*_(2)_ = 0.477; *p* = 0.493] and no interaction effects involving rs2254298 (all *p* > 0.352).

The rs53576 polymorphism had a significant effect on the amount of distress signals children produced in the still phase period [*F*_(2)_ = 5.796; *p* = 0.005], showing that children with the homozygous AA genotype exhibited more distress [M(AA) = 21.52%] than children with the other two genotypes [M(GG) = 7.69%; M(GA) = 7.56%]. There was also a significant interaction effect between experimenter and genotype [*F*_(1, 63)_ = 5.601; *p* = 0.006] showing that this difference was mainly attributable to tests administered by Experimenter 2. When Experimenter 1 administered the test, the amount of distress signals produced showed less variation across genotypes and in general, distress signals were scarcer [M(GG) = 6.88%; M(GA) = 10.59%; M(AA) = 9.55%]. The analyses also replicated the effect of age [*F*_(1)_ = 6.067; *p* = 0.017].

##### Dogs—first reactions to the threatening experimenter

Allele variations at rs8679682 did not have a significant effect on dogs' first reactions to the experimenter [$\chi_{\left(2\right)}^{2}$ = 1.144; *p* = 0.564] (Figure 2). However, sex [$\chi_{\left(2\right)}^{2}$ = 4.511; *p* = 0.034] had a significant modulatory effect, showing that while dogs were more likely to react with looking at or approaching the experimenter without tail wagging than to produce a friendly reaction, this was stronger in the case of males. All other effects were non-significant (*p* > 0.236 at removal)

The analyses on the effects of SNP −213AG yielded a significant main effect of allele variations [$\chi_{\left(2\right)}^{2}$ = 8.383; *p* = 0.015], showing that while dogs with the homozygous A (*N* = 3) or the heterozygous genotype (*N* = 10) all reacted with looking at the experimenter without tail wagging, the behavior of the homozygous GG genotype was more diverse with 11 out of 37 dogs reacting in a friendly way. All other effects were not significant (*p* > 0.086 at removal).

Similarly, SNP−74GC significantly modulated dogs' behavior [$\chi_{\left(2\right)}^{2}$ = 10.861; *p* = 0.004]. While dogs with the heterozygous genotype all (*N* = 13) reacted with looking at the experimenter without tail wagging, participants with the homozygous G genotype also produced friendly reactions (10 out of 29). −94TC polymorphism did not have a significant effect on dogs' first reactions [$\chi_{\left(2\right)}^{2}$ = 3.356; *p* = 0.187].

#### Correspondence between tasks

Children that chose correctly in the first task spent less time (mean: 24.92 s) looking at the experimenter in the *still face* situation than those that could not find the reward [mean: 34.71 s; *t*_(46)_ = 2.37; *p* = 0.022]. The same was true for dogs: those that chose the baited container spent significantly less time looking at the experimenter in the *threatening approach task* [mean: 93.29 vs. 85.39 s; *d*_(49)_ = 2.482; *p* = 0.017]. We found no other associations between performance in the *gaze following task* and the variables coded for the *reaction to an aversive social interaction task*.

## Discussion

The present study explored associations between variation in the OXTR and reaction to an aversive social interaction as well as use of a gaze cue to locate hidden food in dogs and humans. Results seem to support our hypotheses that the oxytocinergic system may play a similar role in shaping dogs' and human infants' reactions to their partner's unexpected negative (distressing) behaviors but not to her gaze cue in a search task. We suggest that this is because the latter is potentially a competitive (and thus distressing) context for dogs, while it would be a cooperative context for human infants.

Our results show that SNP in the gene coding for oxytocin receptor binding are indeed associated with both dogs' and children's reactions to a violation of normal social interactions. We found that dogs' first reactions (either friendly or neutral/fearful) were significantly modulated by two of the four polymorphisms analyzed (−213AG, −74GC). One of these polymorphisms (−213AG) had already been shown to be associated with proximity seeking, a composite measure that included latency to approach the experimenter after the threatening approach task (Kis et al., 2014). Also, it has been shown that intranasal administration of oxytocin influences dogs' reaction in the threatening approach task (Hernádi et al., 2015). In the corresponding analyses with children, we found that the amount of distress signals produced after the withdrawal of positive social stimulation was significantly modulated by one of the three polymorphisms analyzed (rs53576). These results confirm that variation in the oxytocinergic system influences how dogs as well as humans respond to social threat or a socially ambiguous situation (Huber et al., 2005; Hernádi et al., 2015; Kovács et al., 2016)

Analyzing participants' behavior in the *gaze following* task, we found that three out of the four identified polymorphisms (−213AG, −95TC, and −74GC) were connected to whether dogs approached a food location the human experimenter had looked at beforehand. Importantly, two of these three polymorphisms (−213AG, −74GC) were linked to the dogs' friendliness in the *threatening approach* task as well. For example, dogs with the homozygous G genotype at SNP −74GC were not only more likely to search for food using the gaze direction of a human, but were also less threatened by the experimenter in the subsequent task. The same was true for dogs with the homozygous G genotype at −213AG. Although the present study does not allow us to assign specific functions to specific polymorphisms, these consistencies suggest that similar mechanisms regulate dogs' reaction to a clear social threat and to non-ostensive gaze in a food searching context.

In contrast to this, we found no such associations in the case of the toddlers: none of the candidate polymorphisms affected the children's use of communicative human gaze to locate the hidden toy. However, in the same group of infants, we detected a significant association between the subjects' OXTR genotype and the amount of distress the infants displayed in the *still face* task. This suggests different mechanisms underlying dogs' use of non-ostensive and children's use of ostensive gaze. Research in developmental psychology suggests that even younger infants are prepared to follow the gaze of an interactional partner while they ignore similar gaze cues if those are not addressed to them (e.g., Senju and Csibra, 2008). While it has been shown that infants develop an expectation that the direction of ostensive gaze is referential and it delivers generalizable knowledge (Senju et al., 2008), much less research addressed how humans interpret non-ostensive gaze. In contrast to the infants' performance, a number of studies found that without training and extended experimental pre-experiences, dogs follow communicative human gaze only with their gaze but do not approach a food location indicated in this way (Kaminski et al., 2012; Téglás et al., 2012; Duranton et al., 2017). Furthermore, dogs do not only ignore non-ostensive gaze but in fact tend to avoid a food location that another dog or a human has looked at in this way beforehand (Bálint et al., 2015; Duranton et al., 2017). Confirming these results, our findings suggest that dogs perceive such scenarios as competition over food and do not interpret non-ostensive gaze as a cooperative communicative signal that offers food to them. Dogs seem to respond to the context with markedly more social anxiety than children while at the same time it is still possible that they both interpret the non-ostensive gaze cue itself as an intentional cue that indicates the experimenter's interest in this location (Duranton et al., 2017). Further research will have to investigate this latter question.

Nevertheless, in this study we did not find that dogs as a group would avoid a food location indicated with non-ostensive gaze. On the contrary, our results suggest a surprising strong effect of oxytocin on how dogs perceive such a situation. The percentage of dogs following non-ostensive gaze varied very strongly with genotype of the OXTR, with only 20% of the dogs carrying two C alleles on the −94CT choosing the indicated container in contrast to the 80% of the TT dogs doing so.

Although in dogs, both OXTR polymorphisms that were associated with the dogs' reaction to a social threat were also linked to following a gaze cue to a food location, in human infants, we found only one OXTR polymorphism that was associated with reaction to an aversive social situation. Therefore, further studies should look at additional OXTR SNPs to investigate whether they have corresponding associations in the two tasks used here. There certainly are more human SNPs that merit investigation. For example, an association has been found between the rs4686302 SNP and social cognition deficits (e.g., facial emotion recognition) in children with ADHD (Kalyoncu et al., 2017). Especially interesting for dog-human comparisons, this SNP might be functionally similar to the canine rs851376227 located in the last exon of OXTR. Another study (Isgett et al., 2016) revealed that the rs1042778 SNP in the human OXTR gene is associated with gaining positive emotions from loving-kindness training. This particular SNP is located in the 3' UTR that is a region where canine SNPs have also been found.

Interestingly, although we used different gaze cues in children and dogs and we found that only the dogs' gaze following was linked to how they reacted to an aversive social interaction, we found similar associations in dogs and children between their other behaviors in the two tasks. In particular, we found that those participants—both dogs and children—that followed the experimenter's gaze to a hiding location tended to spend less time looking at the experimenter threatening them (in dogs) or looking at them with a still face (in children). At a first sight this seems to suggest that dogs' and children's behavior are guided by similar mechanisms. This might be even correct at the level that participants that are more skilled at utilizing gaze cues may generally be more adept in social situations and, as such, faster to process negative social stimuli as well. Alternatively, it is also possible that gazing during social threat reflects different motivations in dogs and in children and thus, the consistency is only manifested at the behavior level but is not present in the underlying mechanisms. Analyses on the looking times (both in the case of children and dogs) focused on the attention participants paid to the two potential partners in the situation. The caregiver or the owner represented a secure base for participants; therefore looks directed at them can be interpreted as security or information seeking in a negative or ambivalent social situation. Gaze directed at the experimenter can either show fear or curiosity. However, looking at the experimenter in the *still face* task may not only reflect how fearful they perceived the situation, but how much they were interested in re-engaging her in play.

Note that we found that the identity of the experimenter affected the participants' behavior in both species. Despite of the training all experimenters received, this is understandable considering the social nature of the tasks. Most importantly, the genotype of the subjects was not confounded with identity of the experimenter for either SNP, thus the associations between genotype and behavior found in the study cannot be accounted for by an experimenter effect.

Finally, an interesting puzzle in our data concerns the children's generally low success in using gaze direction to locate the hidden object. As children at this age are typically good in following human communicative gaze, we suspect that the procedure we used explains their low success in this study. One could argue that the fact that the experimenter looked back at the children after her gaze cue made it more difficult for the children to remember which container they should choose. If so, we would expect random choices, in contrast to which we found that children had a tendency to choose the empty container. Therefore, we suggest that children's difficulty in locating the toy stems from their immaturity of inhibitory control. Instead of the training trials that we used for the dogs, we wanted to make sure that the toddlers also understood what they would be searching for by allowing them to see the toy inside the box at the non-cued location before the trial (see in procedure). As such, children's execution of action may be strongly biased by the last seen location of the object which may prevent other cognitive abilities (i.e., gaze following) from being exhibited. A similar dissociation between performance in overt behavior and cognitive processing has been documented in other areas of cognitive development as well (e.g., Onishi and Baillargeon, 2005). Importantly, problems with inhibition may also make genotype × behavior associations unobservable. Thus, we cannot discard the hypothesis that similarly to dogs, children's use of communicative cues is affected by OXTR polymorphisms. Further studies using another experimental procedure will have to address this question. However, even if an OXTR genotype × gaze following association is found, our prediction is that this will not be the same association as we found between OXTR genotype and reaction to still face.

In sum, these results support the idea that similarities observed in the overt behavior of dogs and human children may result from different mechanisms. While variations in the OXTR receptor gene affected both species behavior in a negative social situation, we could find corresponding associations in a gaze following task only in dogs. This raises the possibility that for dogs, the two situations are more alike (potentially fear-inducing or competitive) than for human children. Although the aversive social interaction tasks differed between species, the genotype × behavior associations we found were related to the distressing nature of these tasks both in dogs and children. However, while the same polymorphisms modulated the dogs' behavior in the *gaze following* task as well, we found no such consistencies across tasks in the children. We suggest that this is because young children interpret others' object-directed behavior as a learning opportunity (Csibra and Gergely, 2006) and as mostly cooperative, while dogs may view a social partner in a food searching task in a more antagonistic manner. If so, the oxytocin system can facilitate the success of dogs in participating in fundamentally cooperative, communicative interactions by fostering social approach through the reduction of fear responses in social interactions (Huber et al., 2005).

## Author contributions

JT and ZV designed the study; all authors prepared the study material and data acquisition; KO, KK, SY, and DK entered the data and prepared it for statistical analyses; KO and JT analyzed the data; KO, JT, and ZV interpreted the data; ZV and JT obtained funding; KO wrote the first draft of the manuscript; KO, JT, ZV, and AK critically revised the manuscript for important intellectual content. All authors gave final approval of the manuscript version to be published and agreed to be accountable for all aspects of the work in ensuring that questions related to the accuracy or integrity of any part of the work are appropriately investigated and resolved.

### Conflict of interest statement

The authors declare that the research was conducted in the absence of any commercial or financial relationships that could be construed as a potential conflict of interest.

## References

[B1] Bálint A. Faragó T. Meike Z. Lenkei R. Miklósi Á. Pongrácz P. (2015). “Do not choose as I do!”–Dogs avoid the food that is indicated by another dog's gaze in a two-object choice task. Appl. Anim. Behav. Sci. 170, 44–53. 10.1016/j.applanim.2015.06.005

[B2] Bartz J. A. Zaki J. Bolger N. Ochsner K. N. (2011). Social effects of oxytocin in humans: context and person matter. Trends Cogn. Sci. 15, 301–309. 10.1016/j.tics.2011.05.002 21696997

[B3] Bence M. Marx P. Szántai E. Kubinyi E. Rónai Z. Bánlaki Z. (2017). Lessons from the canine OXTR gene: populations, variants and functional aspects. Genes Brain Behav. 16, 427–438. 10.1111/gbb.12356 27860243

[B4] Boyd R. Richerson P. J. (1998). Culture and the Evolutionary Process. Chicago, IL: University of Chicago Press.

[B5] Chen F. S. Johnson S. C. (2012). An oxytocin receptor gene variant predicts attachment anxiety in females and autism-spectrum traits in males. Soc. Psychol. Pers. Sci. 3, 93–99. 10.1177/1948550611410325

[B6] Csibra G. Gergely G. (2006). Social learning and social cognition: the case for pedagogy, in Processes of Change in Brain and Cognitive Development. Attention and Performance, Vol. 21, eds Munakata Y. Johnson M. H. (Oxford: Oxford University Press), 249–274.

[B7] Csibra G. Gergely G. (2011). Natural pedagogy as evolutionary adaptation. Philos. Trans. R. Soc. Lond. B Biol. Sci. 366, 1149–1157. 10.1098/rstb.2010.0319 21357237 PMC3049090

[B8] Domes G. Heinrichs M. Gläscher J. Büchel C. Braus D. F. Herpertz S. C. (2007). Oxytocin attenuates amygdala responses to emotional faces regardless of valence. Biol. Psychiatry 62, 1187–1190. 10.1016/j.biopsych.2007.03.025 17617382

[B9] Duranton C. Range F. Virányi Z. (2017). Do pet dogs (Canis familiaris) follow ostensive and nonostensive human gaze to distant space and to objects? R. Soc. Open Sci. 4:170349. 10.1098/rsos.170349 28791164 PMC5541559

[B10] Futó J. Téglás E. Csibra G. Gergely G. (2010). Communicative function demonstration induces kind-based artifact representation in preverbal infants. Cognition 117, 1–8. 10.1016/j.cognition.2010.06.003 20605019

[B11] Hare B. Brown M. Williamson C. Tomasello M. (2002). The domestication of social cognition in dogs. Science 298, 1634–1636. 10.1126/science.1072702 12446914

[B12] Hare B. Tomasello M. (2005). Human-like social skills in dogs? Trends Cogn. Sci. 9, 439–444. 10.1016/j.tics.2005.07.003 16061417

[B13] Hernádi A. Kis A. Kanizsár O. Tóth K. Miklósi B. Topál J. (2015). Intranasally administered oxytocin affects how dogs (Canis familiaris) react to the threatening approach of their owner and an unfamiliar experimenter. Behav. Process. 119, 1–5. 10.1016/j.beproc.2015.07.001 26165175

[B14] Huber D. Veinante P. Stoop R. (2005). Vasopressin and oxytocin excite distinct neuronal populations in the central amygdala. Science 308, 245–248. 10.1126/science.1105636 15821089

[B15] Isgett S. F. Algoe S. B. Boulton A. J. Way B. M. Fredrickson B. L. (2016). Common variant in OXTR predicts growth in positive emotions from loving-kindness training. Psychoneuroendocrinology 73, 244–251. 10.1016/j.psyneuen.2016.08.010 27543885 PMC5359600

[B16] Israel S. Lerer E. Shalev I. Uzefovsky F. Riebold M. Laiba E.. (2009). The oxytocin receptor (OXTR) contributes to prosocial fund allocations in the dictator game and the social value orientations task. PLoS ONE 4:e5535. 10.1371/journal.pone.0005535 19461999 PMC2680041

[B17] Kalyoncu T. Özbaran B. Köse S. Onay H. (2017). Variation in the oxytocin receptor gene is associated with social cognition and ADHD. J. Attent. Disord. [Epub ahead of print]. 10.1177/1087054717706757 28478728

[B18] Kaminski J. Schulz L. Tomasello M. (2012). How dogs know when communication is intended for them. Dev. Sci. 15, 222–232. 10.1111/j.1467-7687.2011.01120.x 22356178

[B19] Kim H. S. Sherman D. K. Sasaki J. Y. Xu J. Chu T. Q. Ryu C.. (2010). Culture, distress, and oxytocin receptor polymorphism (OXTR) interact to influence emotional support seeking. Proc. Natl. Acad. Sci. U.S.A. 107, 15717–15721. 10.1073/pnas.1010830107 20724662 PMC2936623

[B20] Kirsch P. (2015). Oxytocin in the socioemotional brain: implications for psychiatric disorders. Dialogues Clin. Neurosci. 17, 463–476. 26869847 10.31887/DCNS.2015.17.4/pkirsch PMC4734884

[B21] Kirsch P. Esslinger C. Chen Q. Mier D. Lis S. Siddhanti S.. (2005). Oxytocin modulates neural circuitry for social cognition and fear in humans. J. Neurosci. 25, 11849–11493. 10.1523/JNEUROSCI.3984-05.2005 16339042 PMC6725903

[B22] Kis A. Bence M. Lakatos G. Pergel E. Turcsán B. Pluijmakers J.. (2014). Oxytocin receptor gene polymorphisms are associated with human directed social behavior in dogs (Canis familiaris). PLoS ONE 9:e83993. 10.1371/journal.pone.0083993 24454713 PMC3893090

[B23] Kis A. Ciobica A. Topál J. (2017). The effect of oxytocin on human-directed social behaviour in dogs (Canis familiaris). Horm. Behav. 94, 513–522. 10.1016/j.yhbeh.2017.06.001 28624235

[B24] Kovács K. Kis A. Pogány Á. Koller D. Topál J. (2016). Differential effects of oxytocin on social sensitivity in two distinct breeds of dogs (Canis familiaris) Psychoneuroendocrinology 74, 212–220. 10.1016/j.psyneuen.2016.09.010 27665081

[B25] Lakatos G. Soproni K. Dóka A. Miklósi Á. (2009). A comparative approach to dogs' (Canis familiaris) and human infants' comprehension of various forms of pointing gestures. Anim. Cogn. 12, 621–631. 10.1007/s10071-009-0221-4 19343382

[B26] Li J. Zhao Y. Li R. Broster L. S. Zhou C. Yang S. (2015). Association of oxytocin receptor gene (OXTR) rs53576 polymorphism with sociality: a meta-analysis. PLoS ONE 10:e0131820. 10.1371/journal.pone.0131820 26121678 PMC4488068

[B27] Miklósi Á. Topál J. (2013). What does it take to become “best friends”? Evolutionary changes in canine social competence. Trends Cogn. Sci. 17, 287–294. 10.1016/j.tics.2013.04.005 23643552

[B28] Nave G. Camerer C. McCullough M. (2015). Does oxytocin increase trust in humans? A critical review of research. Persp. Psychol. Sci. 10, 772–789. 10.1177/1745691615600138 26581735

[B29] Neumann I. D. (2002). Involvement of the brain oxytocin system in stress coping: interactions with the hypothalamo-pituitary-adrenal axis. Prog. Brain Res. 139, 147–162. 10.1016/S0079-6123(02)39014-9 12436933

[B30] Oliva J. L. Rault J. L. Appleton B. Lill A. (2015). Oxytocin enhances the appropriate use of human social cues by the domestic dog (Canis familiaris) in an object choice task. Anim. Cogn. 18, 767–775. 10.1007/s10071-015-0843-7 25647172

[B31] Oliva J. L. Wong Y. T. Rault J. L. Appleton B. Lill A. (2016). The oxytocin receptor gene, an integral piece of the evolution of Canis familaris from Canis lupus. Pet Behav. Sci. 2, 1–15. 10.21071/pbs.v0i2.4000

[B32] Onishi K. H. Baillargeon R. (2005). Do 15-month-old infants understand false beliefs? Science 308, 255–258. 10.1126/science.1107621 15821091 PMC3357322

[B33] Ring R. H. Malberg J. E. Potestio L. Ping J. Boikess S. Luo B.. (2006). Anxiolytic-like activity of oxytocin in male mice: behavioral and autonomic evidence, therapeutic implications. Psychopharmacology 185, 218–225. 10.1007/s00213-005-0293-z 16418825

[B34] Rodrigues S. M. Saslow L. R. Garcia N. John O. P. Keltner D. (2009). Oxytocin receptor genetic variation relates to empathy and stress reactivity in humans. Proc. Natl. Acad. Sci. U.S.A. 106, 21437–21441. 10.1073/pnas.0909579106 19934046 PMC2795557

[B35] Senju A. Csibra G. (2008). Gaze following in human infants depends on communicative signals. Curr. Biol. 18, 668–671. 10.1016/j.cub.2008.03.059 18439827

[B36] Senju A. Csibra G. Johnson M. H. (2008). Understanding the referential nature of looking: Infants' preference for object-directed gaze. Cognition 108, 303–319. 10.1016/j.cognition.2008.02.009 18371943

[B37] Sümegi Z. Kis A. Miklósi Á. Topál J. (2014). Why do adult dogs (Canis familiaris) commit the A-not-B search error? J. Comp. Psychol. 128, 21–30. 10.1037/a0033084 23957741

[B38] Tauzin T. Csík A. Kis A. Topál J. (2015). What or where? The meaning of referential human pointing in dogs. J. Comp. Psychol. 129, 334–338. 10.1037/a0039462 26147704

[B39] Téglás E. Gergely A. Kupán K. Miklósi Ã. Topál J. (2012). Dogs' gaze following is tuned to human communicative signals. Curr. Biol. 22, 209–212. 10.1016/j.cub.2011.12.018 22226744

[B40] Thompson R. J. Parker K. J. Hallmayer J. F. Waugh C. E. Gotlib I. H. (2011). Oxytocin receptor gene polymorphism (rs2254298) interacts with familial risk for psychopathology to predict symptoms of depression and anxiety in adolescent girls. Psychoneuroendocrinology 36, 144–147. 10.1016/j.psyneuen.2010.07.003 20708845 PMC2997902

[B41] Topál J. Gergely G. Erdohegyi Á. Csibra G. Miklósi Á. (2009). Differential sensitivity to human communication in dogs, wolves, and human infants. Science 325, 1269–1272. 10.1126/science.1176960 19729660

[B42] Topál J. Gergely G. Miklósi Á. Erdohegyi Á. Csibra G. (2008). Infants' perseverative search errors are induced by pragmatic misinterpretation. Science 321, 1831–1834. 10.1126/science.1161437 18818358

[B43] Topál J. Kis A. Oláh K. (2014). Dogs' sensitivity to human ostensive cues: a unique adaptation, in The Social Dog: Behavior and Cognition, eds Kaminski J. Marshall-Pescini S. (Cambridge, MA: Elsevier; Academic Press), 424, 319–436.

[B44] Tost H. Kolachana B. Hakimi S. Lemaitre H. Verchinski B. A. Mattay V. S.. (2010). A common allele in the oxytocin receptor gene (OXTR) impacts prosocial temperament and human hypothalamic-limbic structure and function. Proc. Natl. Acad. Sci. U.S.A. 107, 13936–13941. 10.1073/pnas.1003296107 20647384 PMC2922278

[B45] Tronick E. Als H. Adamson L. Wise S. Brazelton T. B. (1978). Infants response to entrapment between contradictory messages in face-to-face interaction. J. Am. Acad. Child Adolescent Psychiatry 17, 1–13. 10.1016/S0002-7138(09)62273-1 632477

[B46] Vas J. Topál J. Gácsi M. Miklósi Á. Csányi V. (2005). A friend or an enemy? Dogs' reaction to an unfamiliar person showing behavioural cues of threat and friendliness at different times. Appl. Anim. Behav. Sci. 94, 99–115. 10.1016/j.applanim.2005.02.001

